# Same patient but different worlds: A state-of-the-art review translating best practice psychosocial care from musculoskeletal care to the orthopaedic context

**DOI:** 10.1186/s12891-024-08107-4

**Published:** 2024-12-05

**Authors:** Marie K. March, Katharine E. Roberts

**Affiliations:** 1 Physiotherapy Department, Blacktown Mt Druitt Hospitals, Western Sydney Local Health District, Marcel Cres, Blacktown, NSW 2148 Australia; 2 Sydney School of Health Sciences, Faculty of Medicine and Health, University of Sydney, Camperdown, NSW 2006 Australia

## Abstract

**Background:**

Individuals with chronic musculoskeletal conditions experience persistent pain and disability that has deleterious impacts on physical function, psychological health, social engagement, relationships, and work participation. This impact is greater in people with psychosocial risk factors, and best practice musculoskeletal care recommends a biopsychosocial approach to management. Orthopaedic surgery is often an effective management approach for chronic musculoskeletal conditions, but research has only recently explored the links between differing patient outcomes after orthopaedic surgery and psychosocial risk factors. Implementing biopsychosocial approaches to musculoskeletal care has taken great strides in the primary care setting however, implementation of the biopsychosocial approach in orthopaedic surgery brings complexity as the context changes from primary care to hospital based secondary care. The aim of this review therefore is to explore implementation of psychosocial care in the elective orthopaedic surgery context, informed by evidence in musculoskeletal care.

**Assessment and management of psychosocial factors:**

Several composite screening tools for psychosocial factors or ‘yellow flags’ are recommended for use in primary care for musculoskeletal conditions alongside a comprehensive patient interview. However, in the orthopaedic surgery context, composite measures have focused on discharge destination, and there is not a universal approach to comprehensive patient interview incorporating a biopsychosocial approach. A range of biopsychosocial approaches to musculoskeletal conditions have been developed for the primary care setting, yet few have been explored in the context of orthopaedic surgery.

**Implementation of psychosocial care:**

Implementing best practice psychosocial care into the orthopaedic context has enormous potential for all stakeholders, but several barriers exist at the level of the individual patient and practitioner, workforce, health service and society. We have discussed key considerations for implementation including workforce composition, patient-centred care and shared decision making, health literacy, continuity of care, and consideration of preferences for women and culturally diverse communities.

**Conclusion:**

This review considers current literature exploring implementation of psychosocial care into the orthopaedic surgery context, informed by current research in musculoskeletal care. This presents a critical opportunity for orthopaedic surgery to provide optimised, equitable, high-value, patient-centred care.

## Introduction

Implementing best practice healthcare for persistent musculoskeletal conditions is a “super-wicked problem” with enormous potential impact for individuals, organisations, and health systems [1]. Individuals with chronic musculoskeletal conditions experience persistent pain and disability that adversely impacts mental health [2], decreases activity participation [3, 4], reduces social connections and increases unemployment [3, 5]. Musculoskeletal conditions account for a large proportion of healthcare use and costs, in both primary and secondary care sectors globally [6–8]. These costs continue to increase as the population experiencing musculoskeletal disorders also experience increasing age and multimorbidity [9, 10]. Within the health systems providing musculoskeletal care, elective orthopaedic surgery is embedded as a key management strategy. For most, elective orthopaedic procedures lead to clinically significant, long-term improvements in pain, function, and quality of life, yet one in five people do not achieve these goals in the first year following surgery [11, 12]. Emerging evidence implicates psychosocial factors as key predictors of those at risk of poor recovery, longer hospital stays, and increased costs [13, 14]. However, clinical orthopaedic pathways have yet to adopt psychosocial factors into assessment and recovery protocols. The biopsychosocial model advocates for consideration of how physical, emotional and social factors interact and impact the individual’s health, which is consistent with the World Health Organisation approach to defining and classifying health and well-being across multiple domains [15, 16]. Psychological factors may include formal psychological diagnoses such as anxiety or depression, beliefs regarding self or pain, such as self-efficacy, maladaptive pain cognitions such as catastrophising, or stressful events such as childhood trauma or disaster. Social factors may include expectations and participation in employment, family roles or cultural duties, the physical environment and financial context [17]. Research in persistent musculoskeletal conditions has demonstrated the importance of assessment and management of psychosocial factors in determining outcomes. Given the continuity of patient characteristics between musculoskeletal care and orthopaedic surgery, there are valuable insights to be gained from musculoskeletal research to benefit orthopaedic surgery care. The aim of this review is to explore implementation of psychosocial care in the elective orthopaedic surgery context, informed by evidence in musculoskeletal care.

### Assessment of psychosocial factors

There are a range of biological, psychological, and social factors that predict non-recovery from musculoskeletal pain. Biological factors include age [18], multisite pain [19], the presence of comorbid medical conditions [20] and nociceptive and nociplastic factors [21]. Similarly, these same variables predict longer hospital stays or slower recovery after elective orthopaedic surgery [22, 23]. Psychological factors that predict non-recovery from musculoskeletal pain include somatisation [24], depressive symptoms [25], anxiety [26], pain catastrophising [25], childhood trauma [27], kinesiophobia [28] and low self-efficacy [29]. These patterns are also demonstrated following elective orthopaedic surgery with pre-operative depressive symptoms, anxiety, pain catastrophising, low self-efficacy and somatisation all predicting worse outcomes [1, 13, 30–34]. The range of social factors that impact recovery from musculoskeletal conditions comprises individual, family, and systemic factors. These include patient recovery expectations, which are heavily influenced by socialisation [35], compensation status [36], health literacy [37], socioeconomic status [38] and cultural diversity [39]. This pattern is repeated in the context of orthopaedic surgery across multiple areas [33, 40–43]. A critical factor in orthopaedic surgery is the importance of co-habitation with a caring adult, as this impacts hospital length of stay which is a key performance measure for hospitals [44]. The patient interview is an ideal time for clinicians to use a broad scope initially when considering psychosocial risk factors for an individual patient, which may prompt more formal assessment of specific psychosocial factors when indicated [25, 45].

**Fig. 1:**
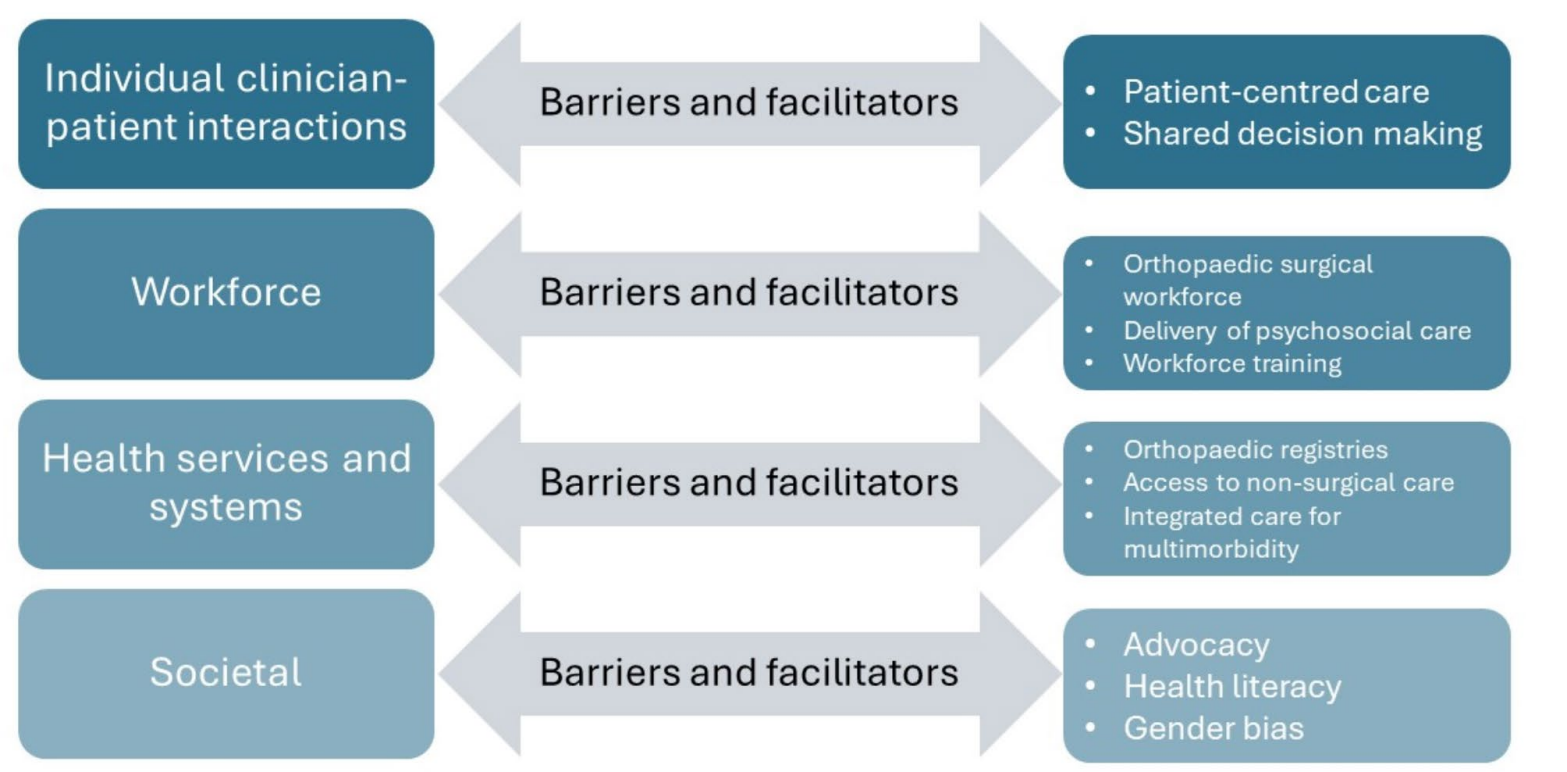
Barriers, facilitators and solutions to implementation of psychosocial care into orthopaedic management

Multiple valid and reliable screening tools are routinely used by clinicians providing musculoskeletal care to assess psychosocial factors. These include composite screening tools such as the STarT Back/STarT MSK or Orebro screening tools [12, 26] and more specific tools such as the Impact of Events Scale, which is recommended in the context of whiplash disorder [46]. For chronic conditions such as knee osteoarthritis, clinicians often assess symptoms of mood disorders such as depression or anxiety [47] as well as self-efficacy, pain catastrophising, or kinesiophobia [48]. In the context of orthopaedic research, assessment of psychosocial factors is more disparate. Studies have re-purposed items from quality-of-life tools as surrogate measures for psychosocial factors, such as the Mental Component Score from the Short-Form Questionnaire which is commonly used. Psychosocial assessment has been incorporated into general prediction tools for lower limb arthroplasty using depression as the sole indicator of psychosocial factors, however these tools lack clinical utility across different contexts [22, 44], or lack sensitivity or consistency across studies [13]. The Depression, Anxiety and Stress Scale, Hospital Anxiety and Depression Scale, and the Pain Catastrophising Scale [30, 49, 50] have also been used in recent research. Psychological resilience is an emerging concept in both musculoskeletal and orthopaedic contexts with the Brief Resilience Scale [51] and Pain Resilience Scale [52] commonly used despite uncertainty regarding the definition and usefulness in the musculoskeletal and orthopaedic contexts [30, 53–56]. Regardless of the psychosocial risk factors measured, there are frequent barriers to implementing assessment in elective orthopaedic care. The barriers, facilitators and solutions that are discussed are outlined in Fig. 1, and need to be applied at the level of the individual clinician-patient interaction, workforce, health services and systems and society.

### Management of psychosocial factors

A range of interventions in clinical musculoskeletal care have incorporated psychosocial elements to facilitate recovery, with early evidence of effectiveness [41, 57, 58]. Current guidelines for managing chronic low back pain recommend biopsychosocial, multidisciplinary management [59], a broad range of interventions incorporating psychosocial components have been developed to optimise management of knee osteoarthritis [58] and psychosocial elements are also used in the management of shoulder pain [60–62]. Evidence from low back pain, knee osteoarthritis and shoulder pain research is most relevant to this review, as recent research has highlighted that some surgical interventions for these conditions may be less effective than first proposed [63].

Research indicates benefits of using psychological approaches in combination with physiotherapy in chronic low back pain [57], and in combination with exercise in knee osteoarthritis [64–66] and shoulder pain [41]. Studies using psychological skills, including mindfulness, cognitive behaviour therapy and pain coping have demonstrated improvements in pain-related activity limitation in low back pain [67], greater efficacy compared to usual approaches in knee osteoarthritis [68–70] and improvements in catastrophising, kinesiophobia and pain intensity in shoulder pain [41]. In an orthopaedic context, there are few robust trials of psychosocial interventions. However, there are promising results for cognitive behaviour therapy to improve post-operative recovery after lumbar spine surgery [71], and mindfulness-based intervention to improve outcomes after knee arthroplasty [68]. Key considerations for further efficacy and implementation research have been highlighted by a recent systematic review, noting greater efficacy for post-operative compared to pre-operative intervention, and the value of psychologist-delivered intervention [72].

### Barriers, facilitators and solutions to implementation of psychosocial care into orthopaedic management

Lin and colleagues outline the components of best practice in musculoskeletal care [25], highlighting the importance of patient-centred care, assessing psychosocial factors and offering high quality, non-surgical care before surgery. Implementation of these principles into clinical practice, as explored in a recent qualitative systematic review [73] can be difficult and slow. Successful implementation of psychosocial care into musculoskeletal services needs consideration at a range of levels: clinician-patient interaction, health workforce, health service and system, and a societal level. However, there are significant challenges in the hospital context that are important when considering implementation in orthopaedic surgery [74–76]. Likewise, implementation of psychosocial care in the context of paediatric and adult cancer care demonstrates the complexity of implementation [77, 78]. Learning from the musculoskeletal care literature may therefore accelerate successful implementation of psychosocial care into the orthopaedic surgery context, which has potential to improve all aspects of the quadruple aim of healthcare: improved patient outcomes, improved patient experience, improved staff experience, improved value [79].

### Individual clinician-patient interactions

#### Patient-centred care

Health systems globally have acknowledged the importance of patient-centred care to provide high quality healthcare. The principles of patient-centred care were first described by Picker [80] and subsequent authors [81, 82], highlighting the eight concepts that define patient-centred care. These concepts include fast access to reliable healthcare, effective treatment by trusted professionals, continuity of care, involvement of family and carers, clear communication and support for self-care, shared decision making, empathy and respect, and attention to physical and environmental needs. These concepts primarily relate to the clinician-patient interaction and overlap with psychosocial care. As such, empowering individual clinicians to be proactive about psychosocial care has great potential for improving surgical outcomes.

In the orthopaedic context, there are many barriers to the implementation of routine psychosocial care at the level of the clinician and patient. Clinicians highlight the need for psychosocial assessment to be accompanied by appropriate referral and management pathways for mental health care for patients who have significant symptoms [83]. Patient expectations of orthopaedic care often do not include psychosocial care, and patients may feel misunderstood, or not believed, in this context [84]. Patients may also feel stigma when psychosocial factors are identified, and decline referral to specific services for these concerns [85, 86]. However, integrating physical and psychosocial approaches to orthopaedic care may in fact, be more acceptable to patients who experience mental health related stigma than to those who do not. Utilising orthopaedic clinicians, within their full scope of practice including physiotherapists, occupational therapists, nurses and social workers, to provide management strategies for both physical and psychosocial health may be one solution [87]. For some populations, improved continuity of care with clinicians and strengthened therapeutic alliance between clinicians and patients may improve both physical and psychosocial outcomes [88]. Implementation strategies to improve therapeutic alliance need to consider how to engage clinicians in this dynamic, reflective process [89, 90].

### Shared decision making

Shared decision making is a core principle of patient-centred care. The exponential increase in demand for elective orthopaedic procedures after COVID19 has increased the impetus for ensuring patients are making informed decisions regarding surgery, and ensuring optimal outcomes [91]. In musculoskeletal care, the importance of the therapeutic alliance to support shared decision making is acknowledged and essential to optimise outcomes [92–94]. However, in orthopaedics, shared decision making has often been reduced to the use of a decision aid, without consideration of the interactive processes required, and unsurprisingly, leading to no improvement in surgical outcomes [95–97]. This is further complicated for patients with cultural and linguistic diversity [98], where development of therapeutic alliance, trust in clinicians and differing risk perceptions, are markedly different compared with white English speakers [99, 100]. Among diverse populations, sociocultural beliefs and past experiences of surgery within their family drive patient perceptions of effectiveness of procedures [101]. However, when patient decision aids factor individual patient risk and are supported with provider training in decision making conversations [102, 103], the quality of decision making, patient expectations and readiness for surgery all improve. Provider training needs to consider role play and simulation approaches that develop therapeutic alliance as well as specific skills regarding shared decision making [104] and cultural responsiveness [105–107].

### Workforce

The orthopaedic workforce have reported many barriers to implementation of routine, formal psychosocial assessment including low acceptability, stigma, lack of time, discomfort, and insufficient communication skills, lack of knowledge, inadequate management support and scarce resources [84, 108–110]. However, orthopaedic workforces have rapidly adopted other emerging approaches, such as robotic surgery and enhanced recovery pathways [111]. Enhanced recovery pathways are multicomponent pathways aimed at optimising all aspects of perioperative surgical care, facilitating a rapid discharge home from hospital [112, 113]. Incorporating psychosocial assessment into enhanced recovery pathways may improve stratification of patients to enhanced recovery or usual care pathways, as patients with psychological comorbidity still experience worse outcomes in the enhanced recovery context [114]. Learning from implementation of other key components of safe, high quality care, such as checklists [115, 116] may provide some insights that can be applied to implementation of psychosocial care. Using evidence-based, intentional change management and implementation pathways for psychosocial care needs to be considered as part of the evolution of best practice orthopaedic surgery.

### Orthopaedic surgeon workforce

Consideration also needs to be given to the specific context of orthopaedic surgery. Orthopaedic surgery is a profession with a predominantly white male demographic, and displays the slowest increase in the inclusion of females and ethnic minorities of any surgical speciality, despite many initiatives to address this [117–120]. Females represent the majority patient group, they access the highest frequency orthopaedic procedures [121, 122], and many females express a preference for female clinicians [123]. Further to this, ethnically diverse minorities who have limited access to both surgical and conservative care options, often report varying surgical outcomes [124], and tend to prefer clinicians who share their ethnicity and/or speak their language [125]. The development of trust between clinician and patient varies in the context of cultural diversity, and increased continuity of care by the same provider, or improved access to clinicians with common language and/or culture may therefore increase trust and improve equity in patient outcomes [126]. Orthopaedic clinicians and researchers should investigate how to identify and address gaps in access and quality of care faced by patients experiencing socio-gendered inequity, similar to previous research in the context of osteoarthritis [127–129].

### Delivery of psychosocial care

Emerging research explores psychological assessment and intervention in the orthopaedic population, however, within this research, these assessments and interventions have been delivered by psychologists or psychiatrists [69, 70, 130, 131]. Despite the existence of large, multidisciplinary teams providing care in orthopaedic surgery, these specialist mental health disciplines are not currently routinely embedded in orthopaedic services [132]. Workforce costs, which may not be recouped by current orthopaedic funding models are a significant barrier to incorporating these disciplines. An alternative approach is to consider which health professionals currently within orthopaedic teams could conduct a comprehensive psychosocial assessment, considering staff time, professional scope, service context, and their existing role within the orthopaedic team. Current evidence describes novel, transdisciplinary interventions developed by nurses, social workers, or physiotherapists, with appropriate training in psychologically informed assessment and intervention [87, 133–135] leveraging the full scope of practice of each profession, minimising costs of additional staff while maintaining continuity of care for patients.

### Training the orthopaedic workforce

Implementation of psychosocial care into ‘business as usual’ orthopaedic care pathways requires a comprehensive approach, including consideration of the training needs and preferences of the existing orthopaedic workforce, supervision and safety requirements, and the development of pathways to access specialist care for high risk mental health patients when required [85, 136]. Although standardisation of processes is helpful in implementing patient-centred care [137], this cannot be at the expense of personalised care. Evaluation of models of care needs to use both process outcomes, such as uptake of psychosocial assessment, alongside traditional outcomes, such as hospital length of stay, patient safety, patient-reported outcome measures and patient experience measures. Future implementation projects need to ensure appropriate representation and contribution from diverse groups in their research to maximise equity of patient experience and outcome [138], as it is these groups (such as females and culturally diverse populations) who stand to benefit the most from an integrated physical and psychological health approach, yet who have been excluded from research previously [139–143].

### Health services and health systems

#### Orthopaedic registries

In addition to individual clinician-patient interaction and workforce concerns, successful implementation needs to consider the health service and health system context where psychosocial care needs to be embedded. An existing strength within orthopaedic surgery is the use of orthopaedic registries, particularly in the elective arthroplasty context. Registries have been successfully integrated into routine clinical care internationally and have provided means to assess prosthesis safety, survival and revision rates [144]. In recent years patient-reported outcome measures such as the Oxford Knee Score, Knee/Hip Injury and Osteoarthritis Outcome Score (KOOS/HOOS) have been added to registry datasets to allow more comprehensive assessment of these important outcomes [144]. Leveraging this unique aspect of orthopaedic care by adding appropriate measures of psychosocial health such as the Depression, Anxiety and Stress Score-21 or Hospital Anxiety and Depression Scale to standardise collection of patient-reported outcomes for registries may empower orthopaedic clinicians to initiate psychological assessment without targeting specific patient groups [108, 109, 121, 145]. Furthermore, recent advancements in electronic data capture and storage can be leveraged to include collection of this data and its integration into existing registries and electronic medical records [146]. The advantage of digital integration presents an opportunity to minimise disparities experienced by people with low literacy or linguistically diverse communities by having a broader range of tools easily accessible [147].

### Access to non-surgical care

The majority of patients seeking elective orthopaedic surgery suffer from chronic musculoskeletal conditions, and evidence indicates that surgical and non-surgical management may yield similar outcomes [63]. However, access to non-surgical care remains low [7, 148]. This lack of access can be attributed to various factors including the beliefs of patients and surgeons, experiences of friends and family, access and funding to non-surgical care [149, 150]. Packages of care based on high quality evidence such as Good Life with Arthritis in Denmark (GLA: D^®^) [151] and the Osteoarthritis Chronic Care Program [152] have been developed to implement non-surgical care more broadly and address the gap in access.

### Integrated care for patients with multimorbidity

Patients experiencing multimorbidity in musculoskeletal and orthopaedic settings often experience both physical and psychological comorbidity, with conditions interacting to worsen each other. Models of care that provide appropriate combinations of care, incorporating physical and psychosocial management, with options to expedite or defer surgery, have demonstrated efficacy and acceptability compared to psychosocial management alone [153, 154], aligning with evidence in low back pain [155]. Despite this, orthopaedic pathways are often focused on standardized, uniform strategies rather than taking a patient-centred or tailored approach such as MOBILISE [112, 156] which is shaped by individual patient goals for behaviour change. Future research needs to consider opportunities for optimising the patient experience and outcomes for diverse populations with physical and psychological multimorbidity [39, 101, 157–160].

### At the societal level

#### Advocacy

Barriers to the implementation of psychosocial care at the societal level include funding and regulation issues as well as health literacy and gender and ethnic biases. Addressing psychosocial care in an equitable way across and within health services needs to be supported at a policy, funding and regulatory level. Advocacy at this societal level for change is best conducted by professional, collaborative and representative groups, which may also serve as communities of practice for clinicians to share knowledge and expertise [161]. Global collaborations such as the Global Alliance for Musculoskeletal Health (gmusc.com) and the International Musculoskeletal Mental and Social Health Consortium (i-mesh.org) provide both communities of practice and advocacy for policy and funding changes, with a view to ensuring equitable global implementation [162]. At a national level, professional licencing bodies and industrial agreements need to support innovative approaches for addressing psychosocial care, such as recognition and renumeration for health professionals using transdisciplinary or extended scope of practice to provide integrated physical and psychosocial care as well as high value care [163, 164].

### Health literacy

Health literacy is a complex concept relating to how people understand and synthesize health information that supports decision making and health behaviours [165]. Health literacy has an important influence on musculoskeletal care outcomes [37, 166] and orthopaedic research is increasingly aware of its influence on surgical outcomes [167–171]. Low health literacy is also a barrier to effective communication and understanding of risk, which is a key aspect of shared decision making. There are opportunities in orthopaedics to tailor existing interventions to address these barriers. For example, education is currently embedded into routine clinical practice, particularly in the elective arthroplasty setting [172, 173], however patient education materials require high levels of English literacy [174]. Utilising effective strategies from musculoskeletal care such as universal health literacy precautions for all educational materials, using non-physician providers, and allowing adequate time for effective interventions in pre-operative pathways [166, 175, 176]. Accuracy of data and use of designs which incorporate a universal health literacy approach [177] may be important in the increasing use of personalised risk profiles [178]. Use of public health strategies to improve orthopaedic health literacy at a societal level such as media-based campaigns [179], and integrating health literacy into adult education settings [180] may also be important approaches to address inequity from a health literacy perspective. Provision of educational materials and measures of psychosocial health in minority languages is also a key opportunity to limit inequity based on language.

### Gender bias

Musculoskeletal and orthopaedic services are embedded in health systems and societies that perpetuate gender and other types of bias affecting females [181]. In the musculoskeletal context, this includes incorrect, non-organic diagnoses [182], inappropriate prescription of anti-depressants for pain [183], and less effective analgesia [184]. Reimagining orthopaedic care to incorporate psychosocial care presents an opportunity to address these inequities. Sex and gender differences in pain coping, pain expression and response to analgesia [184, 185] suggest that models of care tailored to females may be an appropriate way to address inequity of outcome. Given that females are disproportionately represented in orthopaedic surgery populations, development of female-centred models of care may be an approach for future research. Learning from the midwifery profession may be useful as adoption of female-centred, continuity of care models for pregnant women has led to significantly better outcomes and established this model as best practice care [186].

### A call to action

Implementing psychosocial care in orthopaedic care is complex, and there is no universal solution providing assurance of equity of access or outcomes. However, undertaking a structured approach to implementation planning [187], considering the factors highlighted, as well as the local experiences of clinicians and consumers, is a starting point to successful implementation. Future research in this area needs to provide adequate detail regarding local context, population and implementation development processes, in addition to traditional outcomes, so that learning can be applied appropriately by clinicians. Intervention development needs to consider the views from all relevant stakeholders, including implementation scientists, mental health clinicians and researchers, orthopaedic clinicians, health managers and a diverse range of consumers appropriate to the context. Developing models of psychosocial care embedded in orthopaedic health services from inception is essential to evaluate acceptability, effectiveness and sustainability and minimise delayed research translation. Using a cyclical or staged approach [188] to implementation of psychosocial care in orthopaedics is also appropriate given the multi-level, multicomponent nature of implementation.

## Conclusion

Musculoskeletal research demonstrates the value of integrating psychosocial care into physical healthcare to improve patient and health service outcomes. Despite evidence in orthopaedic surgery contexts that demonstrates the association of psychosocial factors with worse outcomes, there are few psychosocial care approaches implemented in orthopaedic surgery contexts. We have explored the barriers and facilitators to implementing psychosocial assessment and treatment in the orthopaedic context, considering implementation at the level individual patient-clinician, workforce, health service and system, as well as at a societal level. Future research needs to explore efficacy and implementation of psychosocial assessment and intervention in the orthopaedic surgery context, with a focus on population groups at high risk of worse outcomes.

## Data Availability

Not applicable.
